# ‘A world of difference’: a qualitative study of medical students’ views on professionalism and the ‘good doctor’

**DOI:** 10.1186/1472-6920-14-77

**Published:** 2014-04-12

**Authors:** Beatriz Cuesta-Briand, Kirsten Auret, Paula Johnson, Denese Playford

**Affiliations:** 1 Rural Clinical School of Western Australia, The University of Western Australia (M706), 35 Stirling Highway, Crawley, Perth, WA 6009, Australia; 2 School of Medicine and Pharmacology, The University of Western Australia (M704), 35 Stirling Highway, Crawley, Perth, WA 6009, Australia

**Keywords:** Professionalism, Medical students’ views, Good doctor, Qualitative study

## Abstract

**Background:**

The importance of professional behaviour has been emphasized in medical school curricula. However, the lack of consensus on what constitutes professionalism poses a challenge to medical educators, who often resort to a negative model of assessment based on the identification of unacceptable behaviour. This paper presents results from a study exploring medical students’ views on professionalism, and reports on students’ constructs of the ‘good’ and the ‘professional’ doctor.

**Methods:**

Data for this qualitative study were collected through focus groups conducted with medical students from one Western Australian university over a period of four years. Students were recruited through unit coordinators and invited to participate in a focus group. De-identified socio-demographic data were obtained through a brief questionnaire. Focus groups were audio-recorded, transcribed and subjected to inductive thematic analysis.

**Results:**

A total of 49 medical students took part in 13 focus groups. Differences between students’ understandings of the ‘good’ and ‘professional’ doctor were observed. Being competent, a good communicator and a good teacher were the main characteristics of the ‘good’ doctor. Professionalism was strongly associated with the adoption of a professional persona; following a code of practice and professional guidelines, and treating others with respect were also associated with the ‘professional’ doctor.

**Conclusions:**

Students felt more connected to the notion of the ‘good’ doctor, and perceived professionalism as an external and imposed construct. When both constructs were seen as acting in opposition, students tended to forgo professionalism in favour of becoming a ‘good’ doctor.

Results suggest that the teaching of professionalism should incorporate more formal reflection on the complexities of medical practice, allowing students and educators to openly explore and articulate any perceived tensions between what is formally taught and what is being observed in clinical practice.

## Background

Medical professionalism is a hot topic [1] and in recent years focus on professional behaviour has increasingly been emphasized in medical school curricula [2,3]. There is general agreement that professionalism is a multidimensional construct [4-7]; however, although definitions of professionalism have been proposed [8], a definite definition remains elusive. Many of the attributes that comprise competence in professionalism have been identified [7,8]. In their review of the literature, Van der Camp and colleagues identified 90 constituent elements of professionalism articulated around three domains of interpersonal, public and intrapersonal professionalism. Altruism, accountability, respect and integrity were the only elements found to be cited relatively often, highlighting the lack of consensus within the medical community [4]. Further complicating the debate, the conceptualisation of professionalism is context-dependent [2,4-6] and is influenced by culturally and socially determined qualities and competencies [9].

A considerable portion of the literature on medical professionalism addresses negative and ‘hidden curriculum’ issues [10-12]. In contrast, few studies have explored students’ perspectives on the constructs of professionalism and the good doctor from a positive viewpoint [5,7,13]. Mounrouxe and colleagues explored explicit discourses on professionalism among students from three medical schools through focus group discussions; the authors identified 19 dimensions of professionalism, and found that discourses varied between pre-clinical and clinical students and also between schools [7]. In their mixed methods exploration of the notion of the ‘good doctor’ among junior and prospective medical students, Maudsley and colleagues [13] found that students valued compassion, patient-centred care and communication skills over clinical competence and knowledge.

The Maudsley study is concerning if professionalism is contrasted with sound medical practice. We therefore sought to explicitly compare students’ views on professionalism and on being a ‘good doctor’. If medical educators are to be successful in nurturing their students’ ‘proto-professionalism’ [8], there is a need to identify discourses which are relevant to students and can be constructively built upon.

The students recruited for this study were in the clinical years (fourth to sixth year) of The University of Western Australia (UWA) MBBS programme. In fourth year, professionalism is taught through a series of lectures and face-to-face meetings with a Personal and Professional Development (PPD) mentor; in fifth year, the PPD program runs throughout the year and is formally assessed through reflective portfolio tasks [14], whilst in sixth year, professionalism is assessed through a case-based ethics essay [15]. A number of those recruited were in the Rural Clinical School of Western Australia (RCSWA) which brings together students from UWA and the University of Notre Dame Australia in a unique clinical school model which has rural health as its base; RCSWA recruits medical students during their fourth year of study through an interview and places them in a rural setting during their fifth year [16].

This paper reports on medical students’ views on professionalism and focuses on students’ perceptions of the constructs of the ‘good’ and the ‘professional’ doctor.

## Methods

As the study was interested in investigating attitudes and perspectives, a qualitative design was chosen; this was suited to the exploratory nature of the study and provided the best fit for the research questions [17], namely, to explore students’ ideas on professionalism and develop terms which are meaningful to them, and to identify barriers and enablers. Data were collected through focus group discussions with medical students; focus groups provide access to a large number of participants, allow for the exploration of group norms and values [18], and have been used to explore medical students’ perspectives on professionalism [5,7]. Data were collected between September 2009 and April 2012; ethics approval was granted by UWA’s Human Research Ethics Committee (reference RA/4/1/2431).

### Sample and recruitment

Medical students in their clinical years (fourth-, fifth- and sixth-year) were invited to take part in the study. Students were recruited through unit coordinators either by e-mail or through personal contact, and invited to take part in a focus group each year throughout their clinical years.

A total of 13 focus groups were held. Five sessions were conducted with fourth-year students, seven with fifth-year students, and one with sixth-year students. The focus groups involved a minimum of two and a maximum of nine students, and had an average duration of 53 minutes.

### Procedure

Focus groups were run by expert facilitators who were neither medical educators nor connected with the medical school. The schedule for the focus group was developed based on a review of the literature; the schedule was semi-structured and, whilst ensuring that all relevant topics were covered in each session, was flexible enough to allow for the introduction and discussion of new topics [18]. Students were invited to share their views on professionalism and the construct of the ‘good doctor’, differences and similarities between the two constructs, barriers and facilitators, and external and internal motivators.

Sessions were conducted in venues easily accessible to students, and sessions involving RCSWA students on placement in regional and remote locations were conducted through video-conference link. All focus groups were audio-recorded. Consent was obtained in writing prior to the focus groups, while verbal consent to record the discussion was obtained prior to each session. Socio-demographic data and background information relating to work experience, religious beliefs, and experience of illness were obtained through a brief questionnaire. All data were de-identified, and data collection continued until saturation occurred [19].

### Data analysis

Focus group discussions were transcribed verbatim and transcripts were subjected to interpretive qualitative data analysis following a grounded theory approach. Data analysis was largely inductive and consisted of thematic analysis based on the four steps identified by Green and colleagues [20]: immersion in the data; coding; creating categories; and identification of themes. A list of codes was developed and reviewed by the research team until consensus was reached; as coding progressed, new codes were created and existing codes refined. Once the coding of the data was completed, connections between codes led to the identification of analytic categories, and ultimately, to the development of an overriding explanation [20]. Data collection and analysis occurred simultaneously, and data collection stopped once data saturation was achieved [21]. Data analysis was aided by the use of the qualitative data software package NVivo 10 [22].

The following techniques were used to increase the trustworthiness of the data [23]: the facilitator who conducted most focus groups performed the data analysis; the coding categories were checked and refined by the research team; data collection and analysis were performed simultaneously; and there was constant referral to the literature. In addition, the use of NVivo 10 enhanced the validity of the results by adding rigour and transparency to the data analysis process [24].

## Results

A total of 49 students took part in the focus groups, 10 of whom participated in two sessions. As shown in Table 1, the majority of participants were female (n = 35) and born in Australia (n = 35). There was equal representation from undergraduate and graduate students (n = 22), and the majority of students were in the 20- to 25-year-old age group (n = 27).

**Table 1:** Sample selected characteristics (n = 49)

**Characteristic**	**Students (n, (%))**
*Sex*	
Female	35 (71.4)
Male	14 (28.6)
*Undergraduate/Graduate*^*#*^	
Undergraduate	22 (50.0)
Graduate	22 (50.0)
*RCSWA Exposure*	
Yes	12 (24.5)
No	37 (75.5)
*Age Group (years)*^*#*^	
20-25	27 (61.4)
26-30	10 (22.7)
31-35	3 (6.8)
36-40	2 (4.5)
41-45	1 (2.3)
46+	1 (2.3)
*Country of Birth*^*#*^	
Australia	35 (79.5)
Other^†^	9 (20.5)

^#^Data available for 44 students. ^†^Malaysia = 2, South Africa = 2, United Kingdom = 4, Zimbabwe = 1.

Results on the constructs of the ‘good’ doctor and the ‘professional’ doctor are presented separately, whilst a final section explores the tensions between the two. All quotes are contextualised by the use of codes identifying the session, year of study and setting.

### The ‘good’ doctor

The ‘good’ doctor emerged as a complex and multifaceted construct; students provided long and articulate descriptions, and they often referred to the notions of ‘balance’ and ‘the art and science of medicine’ in their discussions. Three main themes emerged: competent doctor; good communicator; and good teacher.

#### Competent doctor

Students perceived competence as an essential characteristic of a good doctor, as *‘you can’t be a doctor if you don’t know what you’re talking about’*. In their narratives, clinical competence encompassed possessing academic and clinical knowledge, and applying that knowledge safely. Students spoke at length of the importance of knowledge. However, there was evidence that over the course of their study they increasingly recognised that being aware of one’s limitations was even more critical. Thus, in students’ accounts, self-awareness, humility, and being realistic were perceived as attributes of the good doctor; these attributes stood in sharp contrast to the perceived arrogance of some clinicians who think ‘they know everything’, as the following quote reflects:

*‘A good doctor is one who knows their boundaries. So if they go ‘this is what I know, this is what I don’t know’, so when to be able to refer, when to be able to ask another clinician or look at your textbooks, and actually to be able to be comfortable in themselves to go to their patient when they don’t completely know something, which is not being arrogant and go ‘I know everything’. Like, it’s OK to actually go, ‘well, I don’t actually know that; that’s not my area of expertise’. […] Good academically, good with the patients, and knowing your boundaries for me is a good doctor.’* (FG05, Y5, Rural).

In students’ narratives, a good doctor recognises their own limitations and seeks advice. In contrast, a bad doctor ‘*will just go ahead with something and try and push through’.* Consistent with these understandings, self-improvement and life-long learning were seen as important characteristics of a competent doctor, especially in the context of evidence-based medicine.

#### Good communicator

Good doctors were consistently described as good communicators, and there was evidence that over the course of their medical training, students gained a greater insight into the importance of communication. A student spoke of what it means to be a good doctor:

‘I think it’s a balance of being academically smart and knowing what you’re doing, as well as being able to establish a relationship and rapport with your patients and your peers, because I’ve seen plenty of doctors who can be extremely smart and know everything about their field, but if they can’t establish that rapport with a patient, then the care isn’t as good as it could be.’

Reflecting on how this view had changed over time, the same student commented:

*‘At the beginning of uni it’s all about studying and knowing everything about everything, but as you get into practice into the hospitals, then we can see the importance of actually relating to people around you and establishing those relationships in good solid ways. You see how important that is.’* (FG11, Y5, Urban).

In students’ narratives, good communication with patients and relatives tended to be associated with the notion of ‘connection’ or ‘rapport’, whilst communicating with other health professionals was associated with effectiveness, patient safety and respect.

According to students, good doctors are able to ‘connect’ with patients. This ability to connect with patients was associated with having a holistic approach to medical care and a good bedside manner, and attributes such as friendliness, accessibility, empathy and caring. Students emphasized the importance of two-way interaction; thus, good doctors are able to communicate clearly with patients in a language patients can understand, and they also listen to what is important and relevant to the patient, and learn from patients and families. A fourth-year student commented:

*‘When you’ve had good doctors, they’re not necessarily the ones who come up with the crazy diagnosis, they’re the ones you connect with, and they care about you more, and they can really communicate with you on the appropriate level’.* (FG 10, Y4, Urban).

The comment above alludes to the ability of the good doctor to communicate at the appropriate level; being able to adapt the communication style to suit the specific needs of the patient was commonly perceived as being important, and appeared to have particular relevance for rural students, who were exposed to community practice in small communities and often spoke of their increased awareness of the importance of taking into account patients’ socio-cultural circumstances. The following quote reflects this experience:

*‘I think [a good doctor is] someone who can relate well to their patients, good communicator, and across all levels, being able to change your style of communication and interaction with patients depending on their background and where they come from, say for example, up here we have a lot of Aboriginal patients, so being able to understand culturally where they’re coming from and being culturally sensitive and maybe changing a little of the style of consultation to suit them and make them feel comfortable.*’ (FG8, Y5, Rural).

Taking the time to talk to the patient was perceived to be an essential component of effective communication; however, it was widely acknowledged that time pressures reduced doctors’ ability to communicate with their patients. One sixth-year student reflected on this issue and described herself as a ‘translator’, compensating for doctors’ lack of time:

*‘I think, as a medical student […] I feel like I play the role of the translator. You know, the team will be at the end of the bed, and sometimes I just hang back for a couple of minutes and go, ‘do you understand what’s happening?’ and they’ll go ‘no’, and I’ll quickly try and explain to them, in a language they can understand, what’s going on. Because they’re worried, they’re anxious and so, I guess, in being a good doctor, I want to make sure I don’t ever lose that. Because obviously, you’ll get busier and you’ll have more jobs to do, more to think about, and I understand why doctors don’t communicate to their patients about what’s going on with them, but, not to lose that, that connection to the patient, as you progress through your career, I think is very important.*’ (FG13, Y6, Urban).

With regard to peers and the rest of the medical team, good communication was associated with effectiveness and patient’s safety, as was the notion of interdisciplinary respect. Thus, a good doctor is a doctor who communicates effectively with the rest of the medical team, and treats nursing and allied health staff with respect, acknowledging their contribution to the patient’s care.

#### Good teacher

Being a good teacher was also considered an important attribute of a good doctor; in fact, students referred to this as a ‘duty’ or ‘responsibility’ of the medical profession. Students’ views on what makes a good teacher were strongly influenced by both positive and negative experiences during their clinical placements, and they tended to compare positive role models who take the time to share their knowledge and take an interest in students’ learning to those who are ‘just not interested’.

In students’ narratives, being a good doctor also entailed the duty to be a good teacher to patients, and this teaching role was perceived to be especially relevant in the community setting, where doctors have more opportunities to educate their patients on lifestyle issues and preventive healthcare.

### The ‘professional’ doctor

Many students struggled to articulate their understanding of professionalism, and some admitted to being confused about the meaning of the term. Students’ narratives concerning professionalism were punctuated by pauses and hesitations, and the use of tautological definitions – ‘I see professionalism as professional behaviour’ or ‘acting professionally’ – suggested lack of clarity. Furthermore, their accounts revealed a conflict between acting according to what they understood was expected of them and becoming the kind of doctor they aspired to be.

The main themes emerging from students’ views on professionalism were: the adoption of a professional ‘persona’; acting according to a code of practice and professional guidelines; and treating others with respect.

#### The professional persona

Students widely associated professionalism with the adoption of a ‘professional persona’, which was described as the way in which doctors present themselves to others, including patients, but also colleagues and the rest of the medical team. In students’ narratives, the professional persona was enacted through dressing appropriately and adopting a certain detachment when speaking with patients; both aspects had negative connotations for students and elicited feelings of disdain and scepticism.

Dressing appropriately was a recurrent theme in students’ accounts on professionalism, and there was evidence that this was a part of the formal curriculum which was a source of conflict for students:

*‘When I think about the stuff that we’ve been taught about professional behaviour that I can think of, I can remember being told what we must wear to clinical placements, so certainly our dress. I don’t really remember about being taught how to behave while we’re there necessarily’ .* (FG2, Y4, Urban).

Students appeared to resent being told what to wear. A comment made by a participant in a focus group – *‘a tie makes you perform with higher professionalism’* – elicited laughter among the rest of participants, and suggested feelings of scepticism. Overall, students’ accounts of their perception of the importance of dressing appropriately suggested feelings of disdain towards what they perceived as the ‘superficial face’ of professionalism:

*‘There’s this superficial face that’s put on professionalism in medicine, which is like one doctor said to someone today ‘button up your top shirt, you don’t want to appear too casual’, and I was like well, the difference between this much skin and this much skin, and it’s like to me appearance, that to me doesn’t define professionalism. Professionalism is more about a manner within yourself, and a work ethic, rather than external appearances. And everybody has different personalities, and I don’t think you have to fit into this mould of one specific stereotype doctor’ .* (FG4, Y5, Urban).

As reflected in the quote above, students perceived that they were required to fit into a mould, and resented not being able to maintain their personal style and individuality. Students wished to keep their personal style, and appeared conflicted by the discord between what they were taught and what they witnessed during their clinical placements. This was compounded by students’ perception that patients have different expectations, and so what one patient regards as professional another might view as unprofessional.

Furthermore, adopting a professional persona was associated with a certain detachment in dealing with patients, which came into conflict with the ‘connection with patients’ they perceived to be a characteristic of the good doctor. The following comment highlights this conflict:

*‘Professionalism is kind of this detachment thing, rather than a real… a real connection thing. And that the rapport that you establish is… you know, the idea that I had was that the rapport they teach us to establish is this kind of artificial thing that’s meant to facilitate communication, and it’s a clinical exercise in itself just establishing rapport’ .* (FG2, Y4, Urban).

Students frequently spoke of ‘putting up a show’ according to the requirements of the ‘role’, and one student pointed out that it was possible for students to ‘perform’ according to what was expected for the exams, and then ‘revert back’ to their ways once they graduated. A fifth-year student, reflecting on feedback she had received on a general practice practicum, provided an insight into the conflicting advice students are exposed to during their clinical placements:

*‘My feedback from the GP that I was with was ‘you’re excellent with all the patients’. I was in a really low socioeconomic area, and we were there for eight weeks, so they had lots that came back, and I had really good relationships with them and stuff, and she said ‘you can’t talk to patients like that in the exam because you’ll fail. So you’ve got to be much more distant from them, you’ve got to be much more clinical, you’ve got to be more professional, you can’t say ‘G’day, how are you doing’ when they walk in’. So she was giving me feedback saying that in exams you need to do this, but when you actually practice, it will be really good, just stay like that’ .* (FG4, Y5, Urban).

#### Code of practice and professional guidelines

Professionalism was widely viewed as acting according to codes of practice and professional guidelines, and this domain included the attributes of integrity, respect for patients’ confidentiality and privacy, and being non-judgemental. One fourth-year student reflected:

*‘It’s your code of practice, really. It’s your integrity and the way you act towards not only patients but other professionals you know. Respecting patient confidentiality and privacy and also simple things such as being punctual’ .* (FG01, Y4, Urban).

Rural students appeared to have gained greater insight into the importance of respecting patients’ confidentiality and privacy when practicing in small communities, and they spoke of the challenges they faced as they inevitably became involved in their patients’ private and social lives.

Not crossing boundaries was also associated with maintaining professionalism, and students cited giving out personal mobile numbers to patients as an example of what they perceived as crossing boundaries, and, thus, unprofessional behaviour. Finally, students’ accounts reflected their awareness of the legal implications of failing to comply with professional codes of practice and the importance of adhering to the legal standards, for example, with regard to not having inappropriate relationships with patients.

#### Respect

Treating patients and colleagues with respect was viewed as an important component of medical professionalism, and students’ accounts concerning this issue were influenced by their exposure to clinical role models. When discussing the importance of treating patients and colleagues with respect, students tended to draw on their experience of negative role models; thus, students typically described examples of ‘unacceptable’ or ‘unprofessional’ behaviour they had witnessed in the clinical setting – talking about patients in their presence without acknowledging them, treating patients like ‘specimens’, being rude to nurses and junior doctors, or disregarding the advice of allied health professionals – and subsequently voiced their firm commitment to avoid that behaviour in their own practice. With regard to treating colleagues with respect, students consistently highlighted the importance of interdisciplinary respect, suggesting that this aspect had been formally taught during their medical course.

### Good versus professional doctor

Students’ conflicted views on professionalism came to the fore when they discussed the differences between being a ‘good’ and a ‘professional’ doctor. When asked to compare their understandings of both constructs, opinions varied; however, students tended to think that there was a clear difference between them, as the following quote reflects:

*‘I think there’s a world of difference. I think you can be a professional and you can have a shirt buttoned up to the right thing, and you can have that professional face, and not be good at all’ .* (FG4, Y5, Urban).

Consistent with students’ frequent references to clothes when discussing their understandings of professionalism, students often referred to this ‘superficial side’ to highlight the difference between being a good doctor and acting professionally. Thus, one could be a professional and yet bad doctor by *‘rocking up on time, dressing well, speaking well, not really doing your job, maybe just appearing professional, and not giving the right advice’ .*

Conversely, in students’ narratives, a doctor could be unprofessional, or perceived to be unprofessional, and yet be a very good doctor. Students tended to provide examples of positive role models, highlighting the discord between what students are formally taught and the kind of doctor they aspire to be:

*‘There’s a doctor in [remote town] who swears a lot, and he swears […] in the presence of patients, but he does it in a manner that is very blokey and he gets along with all the miners and he gets along with all the Indigenous blokes, and he does that whole rapport thing really well, which if he was doing that in Perth, I don’t think he’d get away with it. But despite that, he’s probably one of the best practitioners in [remote town] and has great rapport with the majority of the patients, not all, but the majority of patients. And I think he’s not professional at all, but he’s a fantastic doctor. And that really rubbed off on me, that you don’t have to be a lemon to be a good doctor’ .* (FG12, Y5, Rural).

**Figure 1:**
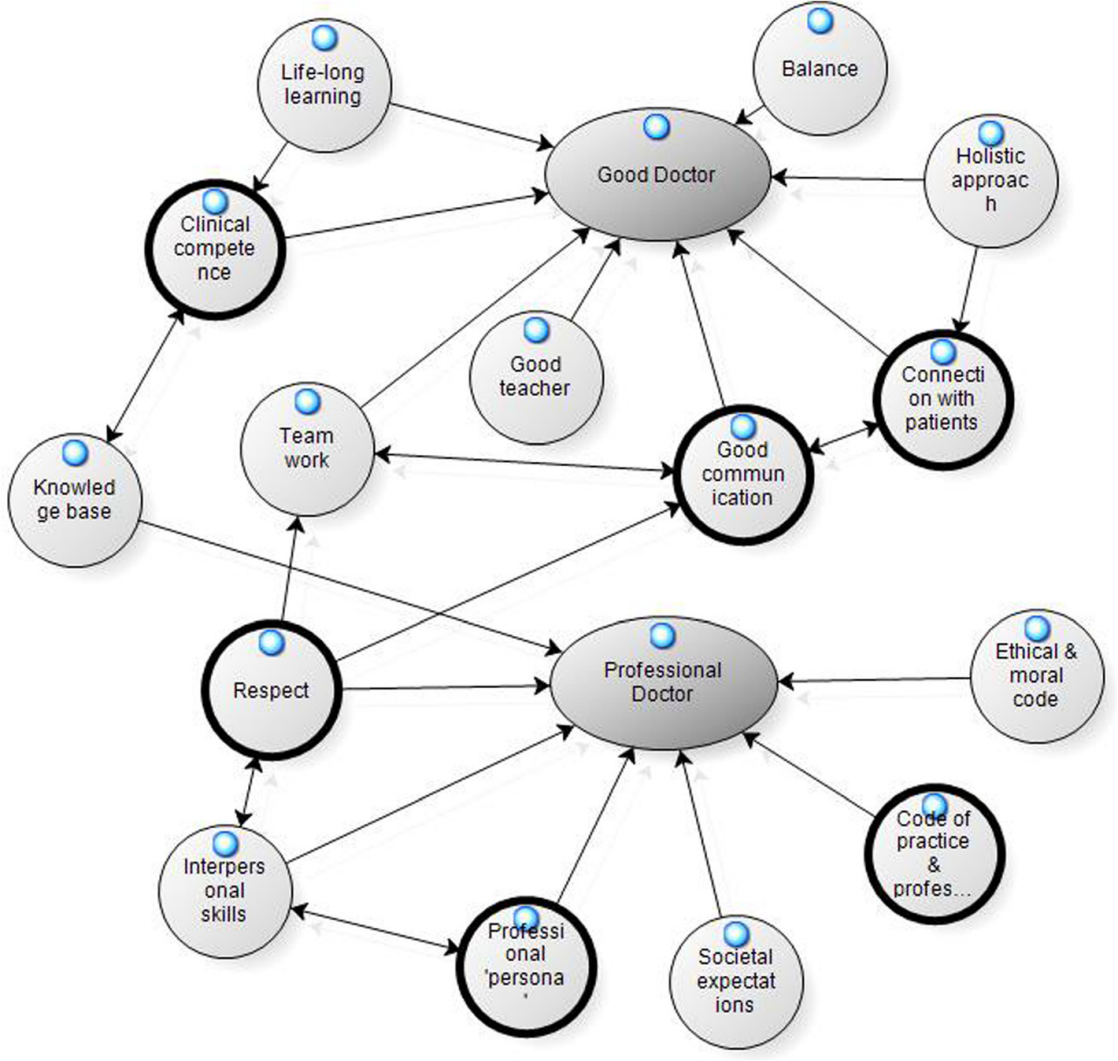
Graphic representation of NVivo coding showing areas of overlap and areas of most coding (bold).

Thus, overall, students tended to describe the ‘good’ doctor and the ‘professional’ doctor as separate constructs. However some overlap was observed, particularly in the domains of respect, team work, communication and knowledge base, as illustrated in Figure 1.

## Discussion

In this study, students perceived ‘good doctor’ and ‘professional doctor’ as two separate constructs with different characteristics and some overlap. Being a good doctor was identified with achieving a balance between the art and science of medicine, between a sound clinical competence and the ability to communicate that knowledge appropriately to patients, relatives and the rest of the medical team. Students’ understandings encompassed elements from canMEDS ‘communicator’ and ‘collaborator’ roles [25], and tended to be aligned with the elements of intrapersonal professionalism described by Van de Camp and colleagues [4]. Somewhat in contrast with findings by Maudsley and colleagues [13], students in our study perceived sound clinical competence as an essential attribute of the good doctor, and their understandings fitted with the ‘3 Cs’ of communication, competence and care that patients seek in a good doctor [26]. Good communication was seen as a core attribute of the good doctor, and was conceptualised as the ability to ‘connect’ with patients and communicate effectively with the rest of the medical team. This result is somewhat consistent with other research conducted with clinicians [27] and medical students [28]; however, in contrast with findings from Bennet and colleagues [28], students in our study did place emphasis on the importance of team work and collaboration.

Professionalism was perceived as an external, imposed construct. Students tended to have one-dimensional views on professionalism and, similarly to participants in Monrouxe and colleagues’ study, they struggled to articulate their understandings [7]. Adopting a ‘professional persona’ was widely associated with professionalism, and the enactment of this ‘persona’ involved dressing appropriately and adopting a certain detachment when dealing with patients, attributes which had negative connotations for students and elicited feelings of scepticism. Consistent with research showing that dressing up is part of ‘switching on’ the professional persona [5], clothing was a recurrent theme in the discussions on professionalism. Students’ perception of the lack of importance of dress standards is problematic, given that evidence shows that doctors’ appearance is important to patients [29,30]. This discord between students’ and patients’ views has implications for PPD education and supports the need to address the importance of appropriate dress standards from a patient perspective.

Students generally viewed professionalism as something that can be activated on demand [5] in order to ‘perform’ as expected, lending support to Brainard and Brislen’s view that students become ‘professional and ethical chameleons’ as a way to navigate medical schools [11]. This finding has implications for medical educators, as it casts doubt on the ability of commonly used assessment items such as Objective Structured Clinical Examination (OSCE) stations or case-based discussions to authentically demonstrate professional behaviour.

In this study, students sometimes perceived the two constructs as acting in opposition, leading to internal conflict on how students perceive situations and feel about themselves and others. Consistent with Bennet and colleagues [28], results from our study suggest that there is conflict between what is being taught and what is being modelled, and there was evidence that students wished to hold on to what they perceived as patient-centred values [12]. There was evidence that students felt more connected to the construct of the ‘good doctor’ – which they perceived as a personally meaningful aspiration – and would forgo professionalism if both constructs came into conflict.

Despite the apparent tension between the construct of professionalism and that of the ‘good doctor’, an area of overlap was observed. Students clearly honoured elements that are core to professionalism, such as respect, team work, communication and having an adequate knowledge base. This finding suggests that these elements, which require internally-motivated behaviour and are associated with both being a professional doctor and a good doctor, should be a starting point upon which medical educators can scaffold discussion about professionalism.

Our findings have curriculum implications, and support the need for greater curricular attention to practical ethics [31]. Our findings suggest that the teaching of professionalism should incorporate more formal reflection on the complexities of medical practice, allowing students and educators to openly explore and articulate any perceived tensions between what is formally taught and what is being observed in clinical practice.

In addition, our findings, which indicate students substantially learn about practice from role models, suggest that identifying clinicians who exemplify what the students most esteem should be a key part of PPD teaching. Mentoring by these clinicians may enable students to integrate what they perceive as the more mechanistic aspects of professionalism with the more competence-based and interpersonal aspects of being a ‘good doctor’. However, given cohort sizes in most institutions and the requirement to rotate students through a number of teaching locations, restricting mentoring to those clinicians identified as exemplifying required behaviours would be impractical. Instead, all mentors could be given assistance in discussing with students how to integrate these topics.

We acknowledge some limitations. Firstly, participants in the study were self-selected, and we cannot discount that they might have been more attuned to ethical and professional dilemmas than the general population of students. Secondly, focus groups may emphasise the stronger voices to the detriment of the weaker ones; in our study, efforts were made to be inclusive and allow all students to express their opinions. Thirdly, only one focus group was conducted with sixth-year students; however, this does not preclude the validity of the data as data saturation was reached.

## Conclusion

In conclusion, this study adds to the relatively scarce literature on the ‘good doctor’ and provides an insight into discourses on professionalism which are meaningful to students and can be constructively built upon. Further research is needed to pilot interventions where the tensions between the two constructs are intentionally explored, and to explore differences between graduate and undergraduate students’ understandings, and between urban and rural students.

## Competing interests

The authors declare that they have no competing interests.

## Authors’ contributions

BCB conducted most focus groups, analysed the data and wrote the initial manuscript draft. KA designed the study and significantly contributed to the pedagogical conceptualisation of the paper. PJ and DP significantly contributed to the study design and the pedagogical conceptualisation of the paper. All authors reviewed and approved the final manuscript.

## Authors’ information

BCB is a qualitative researcher with extensive experience in focus group facilitation. DP is a medical educator at RCSWA. KA and JP are consultant physicians and medical educators at UWA.

## Pre-publication history

The pre-publication history for this paper can be accessed here:

http://www.biomedcentral.com/1472-6920/14/77/prepub
